# The PEGS DREAM Challenge: A Crowdsourcing Approach to Understanding Hypercholesterolemia with Multi- dimensional Genomic and Environmental Data

**DOI:** 10.21203/rs.3.rs-9254914/v1

**Published:** 2026-04-23

**Authors:** Farida S. Akhtari, Johannes Falk, Jyoti Jyoti, Venetia Voutsa, Eda Cakir, Ali Salehzadeh-Yazdi, Marc-Thorsten Hütt, Alessandro Lussana, Federico Marotta, The PEGS DREAM Challenge Community, Geoffrey S. Ginsburg, Gaia Andreoletti, Serghei Mangul, Verena Chung, Jacob Albrecht, David C. Fargo, Charles P. Schmitt, Janet E. Hall, Alison A. Motsinger-Reif

**Affiliations:** National Institute of Environmental Health Sciences; Constructor University; Constructor University; Constructor University; Constructor University; Constructor University; Constructor University; European Bioinformatics Institute; European Molecular Biology Laboratory; National Institutes of Health; Sage Bionetworks; Sage Bionetworks; Sage Bionetworks; Sage Bionetworks; National Institute of Environmental Health Sciences; National Institute of Environmental Health Sciences; National Institute of Environmental Health Sciences; National Institute of Environmental Health Sciences

**Keywords:** DREAM Challenge, Personalized Environment and Genes Study, gene-environment interactions, hypercholesterolemia, high cholesterol, crowdsourced data analysis

## Abstract

Crowdsourced challenges are powerful catalysts for advancing biomedical research and fostering community-driven innovation. The DREAM Challenges initiative, in collaboration with the Personalized Environment and Genes Study (PEGS), held a competition to spur the development of predictive models for hypercholesterolemia risk. Participants were tasked with integrating diverse data, including environmental exposures, whole-genome sequencing, and geospatial information, from a large and diverse cohort to classify hypercholesterolemia and generate novel insights. The top-performing models, which primarily leveraged gradient boosting and random forest classifiers, demonstrated strong predictive performance, outperforming traditional polygenic scores (PGS). In Challenge Task 1, models from the two top-performing teams substantially outperformed the Challenge benchmark dataset using only PGS (AUROC = 0.7358), with AUROCs of 0.7933 and 0.7919, respectively. Beyond prediction, these models highlight the significant value of self-reported health and environmental exposure data, revealing them as informative predictors of hypercholesterolemia risk that complement established clinical and genetic factors. This paper details the design of the challenge, presents the top-performing models, and highlights the potential of integrative, multimodal approaches for understanding and predicting complex human diseases.

## Introduction

Recognizing the profound influence of gene-environment (GxE) interactions on human health and disease, researchers are increasingly integrating genetic and environmental data in studies of etiology. Hypercholesterolemia, commonly called high cholesterol, is a highly prevalent condition and carries a significant risk for cardiovascular disease ^1^. This condition exemplifies the complexity of GxE interactions and their impacts on disease ^2-4^. While genetic predisposition contributes to individual susceptibility to high cholesterol, lifestyle choices, dietary habits, and environmental exposures also influence hypercholesterolemia status.

Traditional approaches often analyze individual data types in isolation, limiting their ability to capture the full spectrum of disease-driving factors. The recent focus on high-dimensional datasets containing multi-omics data, or data from multiple biological systems, is enabling researchers to elucidate the many factors influencing complex conditions such as hypercholesterolemia. For example, incorporating information on environmental factors with genomic data can reveal how risk alleles interact with specific environmental exposures to modulate disease risk. Integrating diverse data types supports a holistic understanding of disease mechanisms, paving the way for more accurate risk prediction, targeted interventions, and personalized medicine.

Dialogue for Reverse Engineering Assessment and Methods (DREAM) Challenges (https://dreamchallenges.org/), in partnership with Sage Bionetworks, are aimed at translating the “wisdom of the crowd” into practice with potentially significant impacts on science and human health. The Challenges engage the scientific community in collaborative problem-solving to address fundamental biomedical issues such as disease classification and prediction using innovative computational models. These global Challenges bring together researchers and data scientists from multiple disciplines to develop and benchmark informatic algorithms. In the DREAM Challenge framework, teams address a specific research question by preparing relevant data and developing models, which are then evaluated and shared. DREAM Challenges have addressed a range of diseases, and results have been published in numerous academic journals ^5^.

**Figure 1:**
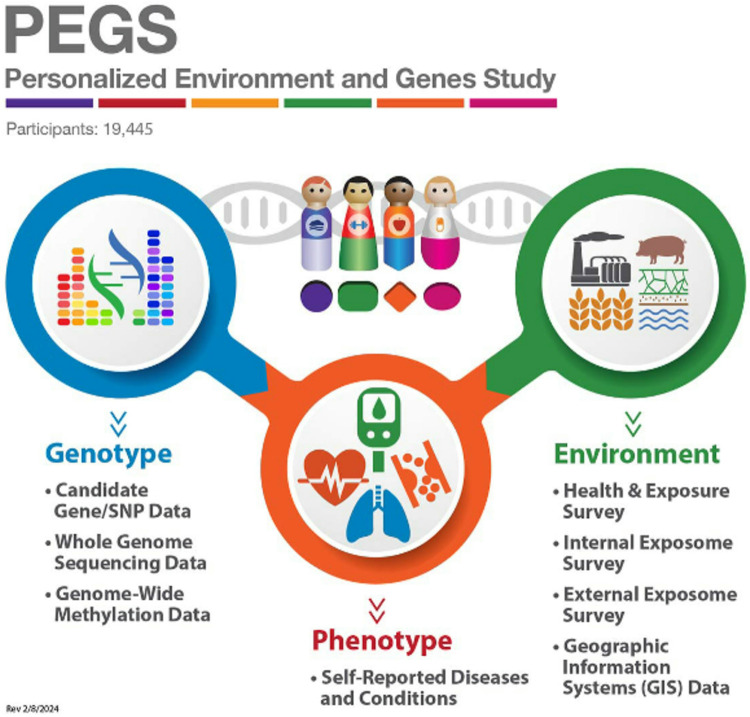
Overview of available PEGS data. Data reported from PEGS Data Freeze 3.1 created on 6/27/2023.

A DREAM Challenge using data from the Personalized Environment and Genes Study (PEGS), a diverse North Carolina-based cohort of nearly 20,000 individuals, was launched to promote innovative statistical and data science approaches integrating multi-dimensional environmental, genomic, and geospatial data to dissect the etiology of hypercholesterolemia (Fig. 1). The PEGS cohort is described in detail on the PEGS website (https://www.niehs.nih.gov/research/atniehs/labs/crb/studies/pegs).

**Figure 2:**
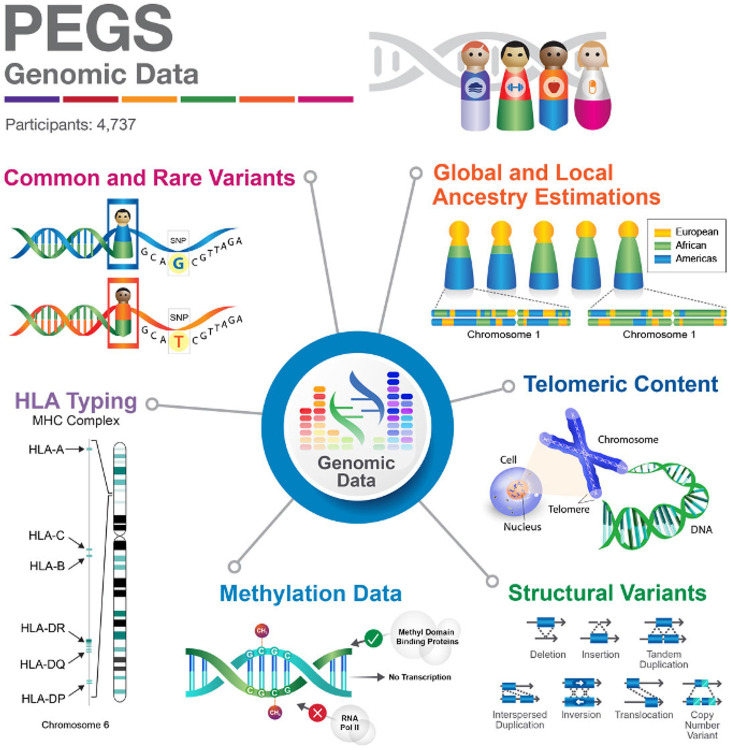
Summary of the genomic data available in the PEGS cohort. Whole-genome sequencing was used to obtain single nucleotide variants consisting of common and rare variants, structural variant calls, human leukocyte antigen (HLA) genotypes for 20 HLA genes, aggregate telomeric content estimation, inferred local ancestry per chromosome, and global estimates of percent ancestry. Genome-wide methylation profiling data were obtained using the Infinium MethylationEPIC v1.0 BeadChip Kit. Data reported from PEGS Data Freeze 3.1 created on 6/27/2023.

Exposomic data gathered through comprehensive surveys capture PEGS participants’ endogenous and exogenous exposures throughout life, including chemical and environmental exposures encountered at work and home, medications, dietary habits, and lifestyle choices. This comprehensive environmental data enables researchers to consider exposures’ cumulative effects and interactions with genomic factors. The whole-genome sequencing (WGS) data available for the PEGS cohort comprises single nucleotide variants (SNVs), structural variants, human leukocyte antigen (HLA) genotypes, telomeric content, ancestry estimations, and genome-wide methylation profiles (Fig. 2), offering insights into both common and rare genetic variations and their potential functional consequences.

**Figure 3:**
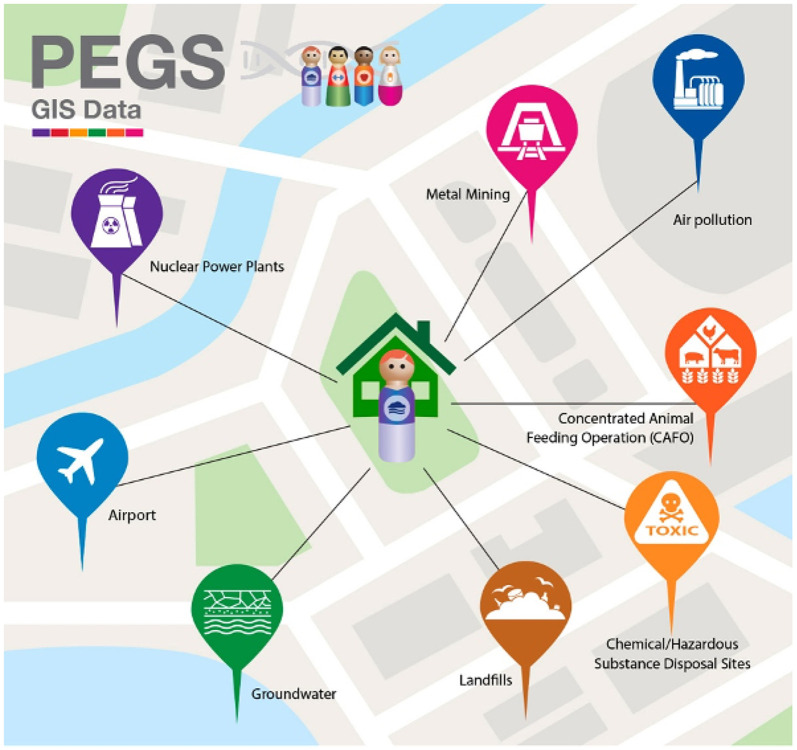
PEGS GIS data. Overview of the multi-pollutant point sources used to estimate exposure indicators for PEGS participants using their geocoded addresses. Data reported from PEGS Data Freeze 3.1 created on 6/27/2023.

Geospatial data were created by linking PEGS participants’ addresses to databases that include proximity to contaminant sources, area-level air pollutant concentrations, and social determinants of health indices such as the CDC/ATSDR Social Vulnerability Index (SVI) (https://www.atsdr.cdc.gov/placeandhealth/svi/index.html) and the Environmental Justice Index (EJI) (https://www.atsdr.cdc.gov/placeandhealth/eji/index.html) (Fig. 3). These indices provide valuable insights into the spatial distribution of environmental risks and their impacts on health disparities.

The PEGS DREAM Challenge (https://www.synapse.org/PEGS) asked participants to develop novel predictive models for hypercholesterolemia by harnessing the power of this multi-omics dataset, with the aim of more accurate disease classification and risk prediction and improved ability to develop targeted interventions and inform personalized medicine. The Challenge featured distinct classification and ideation tasks. In the classification task (Task 1), teams developed models in the multi-omics PEGS data to classify hypercholesterolemia. To assess the potential value of incorporating exposomic and geospatial data for improving classification accuracy, a polygenic score (PGS) derived solely from WGS data was used as a benchmark. For the ideation task (Task 2), teams generated data-driven hypotheses and/or models on the relationships between the multidimensional PEGS data and high cholesterol risk. The ideation task incentivized outside-the-box thinking and promoted collaboration, with the overall aim of unraveling how GxE interactions affect human health.

## Results

### PEGS DREAM Challenge Data

The PEGS DREAM Challenge (https://www.synapse.org/PEGS) asked participants to leverage the multi-dimensional PEGS dataset to identify factors affecting hypercholesterolemia and thus improve understanding of its etiology. The challenge was launched on May 1, 2024, with a Leaderboard round from May 10 to August 6, 2024, and a Final round from August 6 to August 19, 2024. Ninety participants from five continents—Asia, Africa, Europe, North America, and Australia—completed 345 submissions across all rounds of the Challenge. Winners were announced on August 31, 2024 and presented their findings at the Regulatory & Systems Genomics with DREAM Challenges Conference (RSGDREAM2024) in Madison, Wisconsin, USA (https://www.iscb.org/rsgdream2024/home).

Participants took part in one or both tasks:

- **Task 1: Disease Classification:** Classify individual hypercholesterolemia disease status in a held-out test subset containing health, exposomic, geospatial, and genomic data.
- **Task 2: Ideation Challenge:** Develop novel hypotheses and models using the multi-dimensional PEGS data to improve understanding of hypercholesterolemia etiology beyond conventional clinical and genetic risk factors.

Participants were provided questionnaire-based health and exposure, geospatial, and genomic data from PEGS Data Freeze 3.1. Table 4 provides details.

- **Questionnaire-based data:** Demographic, health, environmental exposure, socioeconomic status, and lifestyle data collected from three surveys administered to PEGS participants: the Health & Exposure Survey (N = 9,449), the Internal Exposome Survey (N = 3,071), and the External Exposome Survey (N = 3,618).
- **Genomic data:** WGS data (N=4,737), including SNVs and indel genotypes, structural variant calls, HLA genotypes, aggregate telomeric content, ancestry estimations, and genome-wide methylation profiling data (N=4,724). Methylation profiling was done using the Infinium MethylationEPIC v1.0 BeadChip Kit.
- **Geospatial data:** Exposure estimates and proximity to hazards calculated using geospatial linkages (e.g., proximity to contaminant sources, air pollutant concentrations), Modern Era Retrospective analysis for Research and Applications (MERRA-2) climate/environmental estimates, and SVI and EJI data.

**Figure 4:**
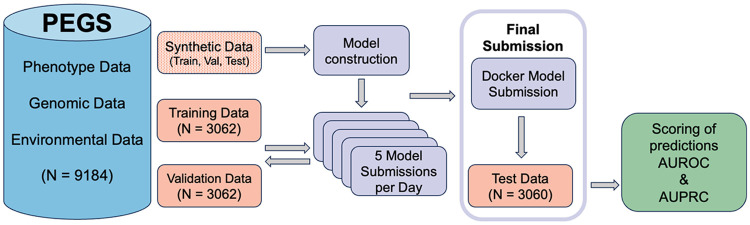
PEGS DREAM Challenge workflow for Task 1. The multi-dimensional PEGS DREAM Challenge data (phenotype, genomic, and environmental data) were randomly and equally split into training, validation, and test data subsets. Synthetic data were available for Challenge participants to download for model construction. Challenge data were split into training (N=3062) and validation (N=3062) subsets. Participants could submit up to five model submissions per day, packaged as Docker containers. The final submitted models were evaluated against a held-out test dataset (N=3060), and their predictive performance was scored using AUROC and AUPRC.

The Challenge data (N = 9,449) were filtered to remove outliers, related individuals, and race mismatches, resulting in a dataset comprising 9,184 individuals. The filtered data were split into individuals with and without WGS data. Each subset was then randomly split into thirds to create training (n = 3,062), validation (n = 3,062), and test (n = 3,060) subsets. Each subset comprised approximately 50% individuals with sequencing data and 50% without sequencing data. The test subset was not shared with Challenge participants and was held out to score Final round submissions. Figure 4 shows the workflow for Task 1.

To protect the privacy of PEGS participants, the original PEGS data were not provided to Challenge participants. Synthetic data, created using the synthpop R library, was provided for model construction and code development. The synthetic data mirrored the structure and sample sizes of the original dataset but did not preserve inter-table correlations. Submitted models were evaluated with the original PEGS data. Privacy measures included de-identification and anonymization with generated IDs in addition to limiting access to original data.

#### Challenge Task 1: Disease Classification

In Task 1, participants developed models in the training and validation datasets to classify hypercholesterolemia status in the held-out test dataset. Participants were encouraged to surpass a benchmark area under the receiver operating characteristic curve (AUROC) of 0.7358, obtained from a hypercholesterolemia PGS. This benchmark PGS was computed for PEGS participants using weights and metadata from the hypercholesterolemia PGS by Weissbrod et al. ^6^ in the Polygenic Score Catalog ^7,8^. This PGS was selected due to its inclusion of multiple ancestries and a large number of genomic variants. Model accuracy was assessed using AUROC, with area under the precision-recall curve (AUPRC) as a tiebreaking metric. Teams Spider Bobs (AUROC = 0.7933) and Nonsense-Mediated Decay (AUROC = 0.7919) submitted the top-performing submissions for Task 1.

##### Team Spider Bobs (Task 1)

Team Spider Bobs integrated data from the Health & Exposure Survey and Internal Exposome Survey using random forest and gradient boosting classifiers from Python’s scikit-learn library ^9^. For data processing, some variables were manually preprocessed. For ordinal variables, coded responses were reordered to reflect ordinality (e.g., Health & Exposure Survey - current physical health rating compared to five years ago as better, worse, or about the same). For continuous (height, weight, age, BMI) and some ordinal (Health & Exposure Survey questions in the fatigue section) variables, missing responses were imputed with the mean value. For skipped questions (e.g., smoking details for non-smokers), missing responses were replaced with appropriate values (e.g., 0). Gradient boosting had better performance on the team’s cleaned data and was used for the final model. The team optimized hyperparameters using cross-validation. Reducing the number of features and sub-samples significantly improved predictions.

**Figure 5:**
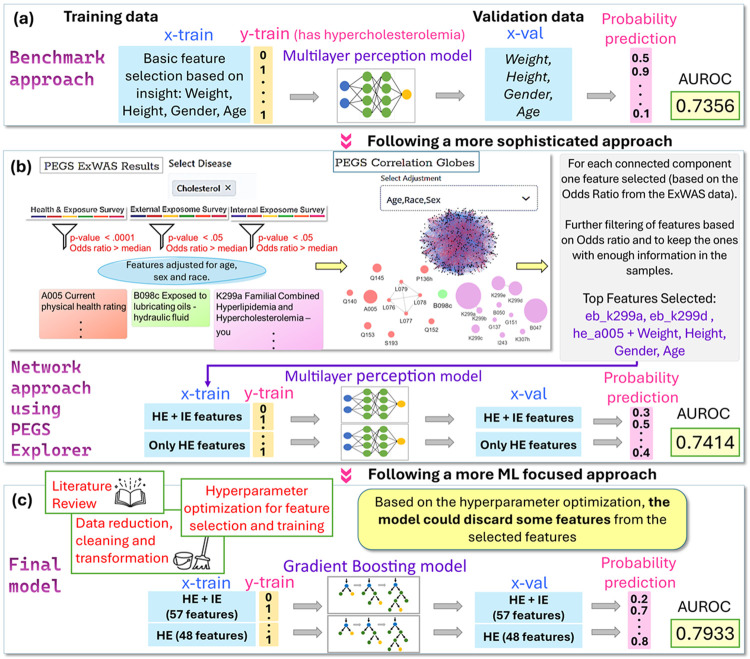
Team Spider Bobs’ workflow for Task 1 – Classification challenge. (a) Benchmark model: Key features were selected to train a multi-layer perceptron (MLP). (b) A correlation network analysis approach was used to identify the most important features in the Health & Exposure, Internal Exposome, and External Exposome Survey data: i. Feature selection was based on *P*-values and odds ratios from PEGS ExWAS results. ii. Correlations among features were obtained from PEGS correlation globes, and a correlation network was created (nodes = features, edges = correlation value). iii. Subnetworks were created from the correlation network for each survey with features selected from PEGS ExWAS results. For correlations, edges > 0.6 were filtered. From each subnetwork, one representative feature was selected from each connected component (including isolated nodes). For model training, three of these features were eventually selected in addition to the features included in the benchmark model. In each case, features with a higher odds ratio were selected. Due to data availability, the model was divided into two parts. Two MLP classifiers were trained on the two data subsets and used for disease prediction (AUROC = 0.7412). (c) Final model: Features with the highest significance were selected based on a literature review, data processing, and hyperparameter optimization. Two gradient boosting classifiers were used for training due to data availability.

Team Spider Bobs used the following workflow (Figure 5):

1. **Benchmark Model** (Figure 5a): An initial model using key features from the Health & Exposure survey (weight, height, sex, age) and trained with a multi-layer perceptron (MLP) had an AUROC of 0.7356 in the validation data.
2. **Refinement** (Figure 5b): The model was refined by leveraging PEGS exposome-wide association studies (ExWAS) results and correlation globes (https://pegsexplorer.niehs.nih.gov) to identify relevant features based on *P*-values and correlations ^10,11^. Features were selected based on ExWAS *P*-values and odds ratios, and correlations were checked using correlation globes. For each survey data component outlined in Table 1 (except demographic and administrative data), features were connected if the correlation value was > 0.6, and a representative feature from each component was selected (Figure 5). These were further reduced to three main features plus benchmark features to mitigate overfitting. Data were partitioned based on the availability of Internal Exposome Survey data, and two MLP models were trained and applied. This refined approach improved the AUROC to 0.7412.
3. **Final Model** (Figure 5c): For feature selection to identify the most significant features for the final model, a literature review, data processing, and hyperparameter optimization were utilized. Data were split again, and two gradient boosting classifiers were trained.

##### Team Nonsense-Mediated Decay (Task 1)

Team Nonsense-Mediated Decay integrated Health & Exposure Survey and genetic data to enhance classification accuracy. Decision trees and ensemble methods such as random forest and XGBoost were used to capture non-linear relationships between mixed data types (e.g., numerical and categorical variables) and perform feature selection. For genomic data, PGS were utilized to complement the random forest model and capture the combinations of variants influencing disease risk. Data were processed to limit analysis to numeric and categorical variables from the Health & Exposure Survey. Variables with more than 5% missing values were removed; remaining missing data were imputed using the median for numeric data and mode for categorical data.

**Figure 6:**
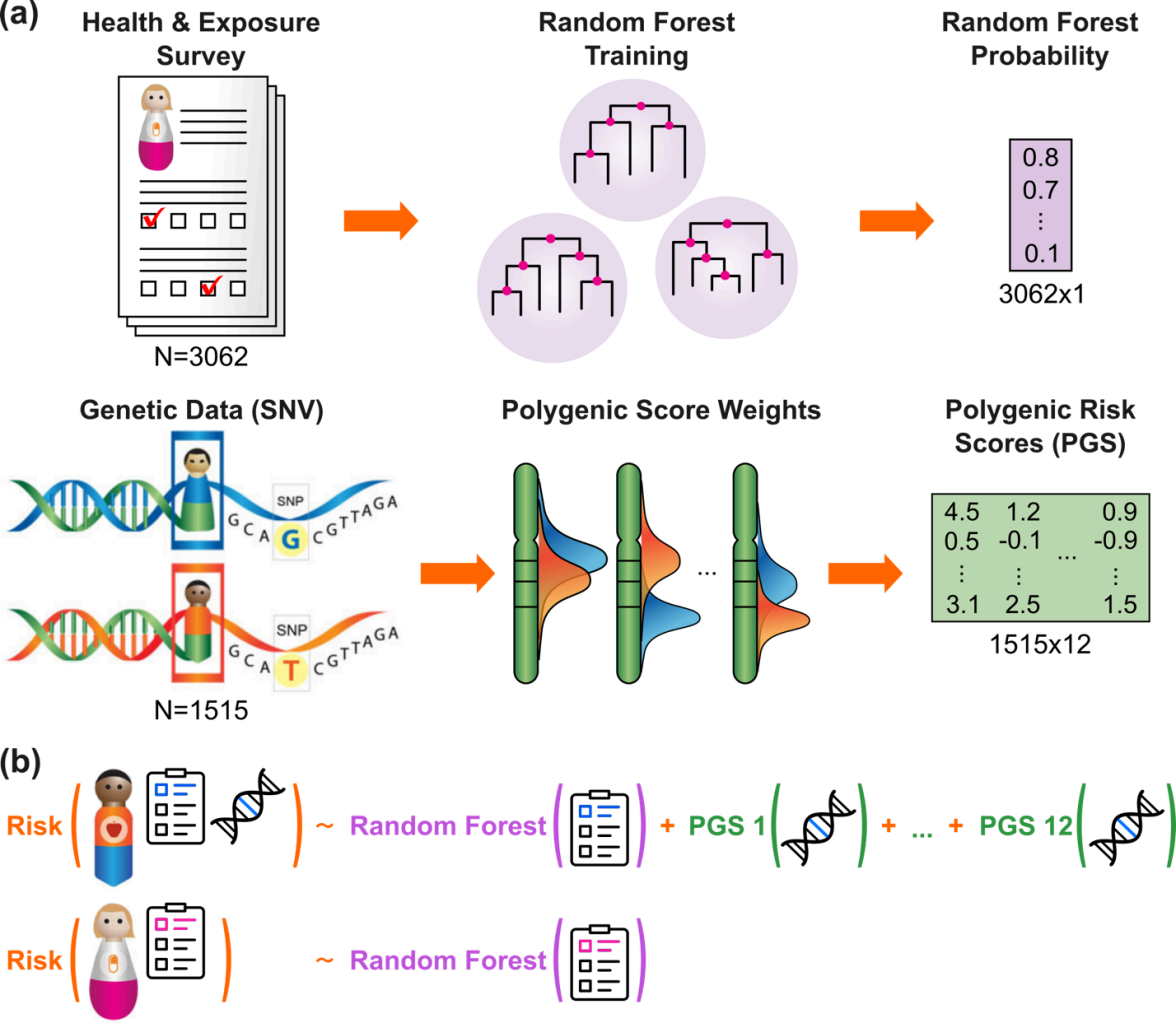
Team Nonsense-Mediated Decay’s model for Task 1– Classification challenge. The hybrid model integrated predictors from both genomic and survey data. (a) A random forest classifier was used for model training with Health & Exposure Survey data to determine hypercholesterolemia probability. For individuals with whole-genome sequencing (WGS) data, 12 polygenic scores (PGS) associated with hypercholesterolemia were calculated using harmonized weight sets from the PGS Catalog.

Team Nonsense-Mediated Decay used the following workflow for a random forest classifier with Health & Exposure Survey data combined with PGS: (Figure 6):

1. **Benchmark Model:** An initial model was trained with a random forest classifier using only Health & Exposure Survey data to estimate baseline probabilities of hypercholesterolemia status, leveraging the model’s inherent feature selection.
2. **Refinement:** Twelve PGS associated with hypercholesterolemia were calculated for PEGS participants with genetic data using harmonized weight sets from the PGS Catalog ^7^. These PGS and the baseline random forest predictions were combined using logistic regression to refine individual disease probabilities.
3. **Final Model:** The final probability of hypercholesterolemia for each individual k was computed using the formula:

P^(Yk)={Xk,bifklacks genetic data,11+e−(β0+βbXk,b+βg1Xk,g1+⋯+βg12Xk,g12)ifkhas genetic data}

where $P(Y_{k})$ is the predicted probability of hypercholesterolemia for individual k, $X_{k,b}$ is the baseline probability estimated by the random forest model, $X_{k,\text{gi}}$ is the *i*-th PGS for individual k, and β represents the logistic regression coefficients. This two-step integration optimized and regularized the weight of genetic and survey predictors. The hybrid model achieved improved classification performance (AUROC=0.775 on the validation dataset) with respect to its individual components using survey data or genetic data only (with AUROCs of 0.758 and 0.736, respectively), demonstrating the utility of combining multimodal data. By incorporating PGS, the model leveraged insights from previous large-scale studies to interpret genome-wide variation.

### Challenge Task 2: Ideation Challenge

In Task 2, participants leveraged the multi-dimensional PEGS dataset to develop novel models and hypotheses with the aim of improving understanding of hypercholesterolemia etiology beyond conventional genetic and clinical factors. Judges scored the hypotheses and models based on creativity, significance, interpretability, innovation, utility, feasibility, and potential translational impact. As with Task 1, the top-performing submissions for Task 2 were from Teams **Spider Bobs** and **Nonsense-Mediated Decay**.

#### Team Spider Bobs (Task 2)

For Task 2, Team Spider Bobs conducted follow-up analyses from their Task 1 model to identify novel factors influencing hypercholesterolemia risk. Methylation data were analyzed using the *limma* R package ^12^ to detect significant differentially methylated probes (adjusted *P*-value < 0.05) for individuals with and without hypercholesterolemia. The results revealed 10 probes (seven hyper, three hypo) corresponding to three hypermethylated genes (*ELOVL2, FHL2, ZYG11A*) and two hypomethylated genes (*PXN, CCDC102B*). *ELOVL2* is involved in fatty acid elongation, potentially influencing lipid metabolism, and *FHL2* has been linked with cardiovascular disease.

Next, association analyses were performed for the HLA genotype data using PLINK’s ^13^ logistic regression model with quality control steps that included removing samples/SNPs with excessive missing data, rare variants, and deviations from Hardy-Weinberg equilibrium. There was a significant difference in the distribution of *HLA-DPB1* alleles between individuals with and without hypercholesterolemia (χ^2^ = 60.01, p = 0.0072). This association remained significant after multiple testing correction. Marginally significant associations with *HLA-H* (*P* = 0.0534) and *HLA-DMA* (*P* = 0.0320) were also observed. There is a known association between familial hypercholesterolemia and certain HLA alleles, and elevated total cholesterol has been correlated with specific HLA variants.

Additional analysis of features included in the gradient boosting model developed for Task 1 revealed that, in addition to well-known risk factors (e.g., BMI, age, smoking, hypertension, alcohol, poor diet, diabetes), factors related to secondary hypercholesterolemia (i.e., high cholesterol triggered by other diseases), such as uterine tumors, also played an important role. The team identified three novel features significantly associated with hypercholesterolemia status in the PEGS data:

- Vitamin E taken regularly in the past year (from the Internal Exposome Survey)
- Regular exposure to dyes (from the Health & Exposure Survey)
- Ever diagnosed with German Measles (from the Internal Exposome Survey)

Using the Informatics for Integrating Biology and the Bedside (i2b2) self-service web-based tool for data exploration (see details in the Methods), Team Spider Bobs further examined these associations. For exposure to dyes, the team hypothesized a correlation with a low standard of living, a known factor for hypercholesterolemia (i.e., workers in chemical/textile manufacturing may earn below-average wages).

Surprisingly, the correlation between diagnosis with German Measles and hypercholesterolemia status had an extremely small *P*-value (p ≈ 6.7e-32). An interaction between the German Measles vaccine and the *HLA-DPB1* gene has been reported. A small number of individuals fail to build protective antibodies despite vaccination, and differences in immunity to the Rubella virus have been associated with the *HLA-DPB1* gene^14,15^. Based on the observed correlations in PEGS data and improved model scores in Task 1 when including ‘Ever diagnosed with German Measles’, the team hypothesized that infection with German Measles is associated with hypercholesterolemia status in the PEGS data.

To test the hypothesis accurately, the team suggested using German Measles antibody results for PEGS participants, as self-reported diagnoses are prone to error (around half of infections go undetected)^16^. This antibody data would enable further testing of the association of hypercholesterolemia and *HLA-DPB1*. However, the team acknowledges that this putative association could be due to the potential correlation of German Measles and hypercholesterolemia with socioeconomic factors.

#### Team Nonsense-Mediated Decay (Task 2)

For Task 2, Team Nonsense-Mediated Decay proposed a GxE interaction model that integrates genomic data on SNVs with Health & Exposure Survey data. These components were selected due to their broad availability in the cohort and their observed utility in improving Task 1 classification performance.

Data processing steps include the following. SNV genotypes are encoded based on alternative allele dosage (0, 1, 2). To ensure statistical robustness, only SNVs with a minor allele frequency > 0.05 are considered. Population structure is accounted for with principal component analysis (PCA) of the genotype matrix, with top components included as covariates. Health & Exposure survey responses are filtered, and variables with more than 5% missing responses and more than 95% single response frequency are removed. Variables with strong collinearity or sex-dependent responses are also removed. Numerical and ordered categorical variables are used as-is; binary variables were encoded as 0/1; unordered categorical and free-text variables were excluded.

The marginal effect a of a given SNV (G) on trait (Y) is first quantified as:

$$Y=a_{0}+a_{1}G+\in$$

where $a_{0}$ is the intercept and ∈ is the error term.

To estimate the genetic main effect $b_{1}$, in the presence of the environment (E), the model can be extended to:

$$Y=\beta_{0}+\beta_{1}G+\beta_{2}E+\beta_{3}(G\;x\;E)+\in$$

where $b_{2}$ represents the environmental main effect, and $b_{3}$ the interaction effect. Covariates from PCA and other relevant confounders such as sex are included in the models to account for population structure and confounding.

To detect significant GxE interactions, the linear models described above can be fit to all selected G,E pairs. The relationship between the genetic marginal effects ($a_{1}$) can then be modeled in the absence of E and the genetic main effects ($b_{1}$) in the presence of E with linear regression. As previously demonstrated ^17^, in the absence of a true GxE interaction effect, $b_{1}$ and $a_{1}$ are linearly correlated. This developed hypothesis leverages this property to systematically test for the presence of GxE interactions in the data. Potentially interacting G,E pairs can be ranked based on their deviation from this expected correlation and reviewed for biological interpretation.

## Discussion

The PEGS DREAM Challenge demonstrates the power of crowdsourced problem-solving in leveraging multidimensional data to investigate the etiology of a complex condition—hypercholesterolemia. This initiative brought together diverse expertise from across the world to develop integrative models and testable hypotheses, yielding both methodological innovation and new biological insights. The models presented in the PEGS DREAM Challenge demonstrate that integrative approaches combining self-reported exposome and health survey-based data with genomic information can outperform models based on PGS alone. This finding underscores the value of incorporating multi-dimensional data, such as those in the PEGS cohort, to capture the complex interplay of genetic and environmental factors in diseases like hypercholesterolemia. This Challenge exemplifies how community engagement in a competitive yet collaborative setting can accelerate methodological advances and generate new research directions in precision environmental health.

In Challenge Task 1, the top-performing teams substantially outperformed the benchmark PGS (AUROC = 0.7358), with AUROCs of 0.7933 and 0.7919, respectively, highlighting the usefulness of integrating survey-based health and exposure data with genomic information. Team Spider Bobs relied on correlation networks derived from ExWAS results to identify informative features, while Team Nonsense-Mediated Decay combined ensemble learning with PGS to build a hybrid model. The comparable performance of these two methodologically distinct approaches demonstrates that robust prediction of hypercholesterolemia status can be achieved using flexible combinations of multimodal data. Importantly, these models show that self-reported health and exposure variables—often underutilized in clinical prediction—add measurable value to disease classification beyond genetic risk scores.

Challenge Task 2 extended the utility of the PEGS dataset beyond classification, asking participants to generate novel hypotheses regarding hypercholesterolemia risk. These ideation submissions revealed potential biological mechanisms and environmental contributors that warrant further investigation. For example, Team Spider Bobs identified differentially methylated probes in genes such as *ELOVL2* and *FHL2*, implicating lipid metabolism and cardiovascular regulation pathways. Their finding of a significant association between HLA-DPB1 alleles and hypercholesterolemia echoes known links between HLA variation and lipid phenotypes, suggesting immune-mediated contributions to metabolic disease risk. Unexpected associations of hypercholesterolemia with self-reported exposure to dyes and diagnosis with German Measles also emerged. Although these findings may reflect latent confounding (e.g., socioeconomic status), they illustrate how unconventional variables can surface through hypothesis-free exploration and generate new research questions. Team Nonsense-Mediated Decay, meanwhile, proposed a systematic method for detecting GxE interactions using deviations from expected genotype-phenotype correlations in the presence of environmental variables. Their approach, rooted in modeling marginal versus conditional genetic effects, highlights the utility of theoretical expectations to identify non-additive interactions. This strategy is broadly applicable to other traits and datasets, offering a reproducible framework for large-scale GxE analysis.

Beyond the findings themselves, the Challenge showcased key methodological innovations. The use of synthetic data allowed teams to develop and refine models while maintaining participant privacy. The provision of resources such as PEGS correlation globes and ExWAS results enabled participants to perform informed feature selection and contextual interpretation. These tools, combined with Docker-based model evaluation, ensured reproducibility and transparency in model scoring, setting a standard for future data science competitions involving sensitive health data.

## Conclusions

Ultimately, the PEGS DREAM Challenge fostered a fertile environment for developing new approaches to integrating genomic, environmental, and social determinants of health data. The top-performing models offered not only improved classification tools but also a roadmap for incorporating diverse data types into disease modeling. These models could be clinically relevant and help build robust and translatable predictive tools for personalized medicine with validation against objective clinical outcomes using data such as electronic health records. The ideation task reinforced the importance of hypothesis generation in complex trait research, particularly when guided by novel, interpretable, and potentially actionable variables. Taken together, the results of the Challenge suggest that integrative, crowdsourced approaches can reveal both methodological and biological insights into diseases shaped by GxE, which will be critical as biobank-scale datasets continue to expand.

## Methods

### PEGS DREAM Challenge Data

#### Personalized Environment and Genes Study (PEGS) cohort

Originally established in 2002 as the Environmental Polymorphisms Registry (EPR) to recruit participants for ongoing research at NIEHS by convenience sampling at community events, PEGS was renamed in 2022. This racially and ethnically diverse North Carolina-based cohort is a repository of data on medications, health outcomes, environmental exposures, lifestyle factors, genomic data, and geospatial estimates of exposure (Figure 1). PEGS participants complete three surveys—the Health & Exposure Survey and the Internal and External Exposome Surveys—and provide biological samples. Participants can consent to be called back for additional tissue collection for add-on studies. Further details of PEGS can be found at https://www.niehs.nih.gov/research/atniehs/labs/crb/studies/pegs/index.cfm. In contrast to typical studies that focus on a single disease or environmental exposure, PEGS gathers data on a wide array of diseases and environmental exposures, including dietary and lifestyle factors, in conjunction with genomic data. The overarching aim of PEGS is to empower researchers to unravel the etiology of disease and uncover how environmental, dietary, lifestyle, and genetic factors collectively impact human health.

The PEGS cohort (N=19,445) comprises approximately two-thirds female (62.3%; 12,120) and one-third male (37.6%; 7,298) participants ranging in age from 18.4 to 98.3 years, with a mean age of 50.2 years (at completion of the Health & Exposure Survey). The self-reported racial makeup of the cohort is two-thirds White (63%; 12,279), slightly over one-quarter Black (27.6%; 5,361), and 4.5% who identify as another race (868). Additionally, 5.0% self-reported their ethnicity as Hispanic (979). This diverse cohort includes participants of varying socioeconomic status and education levels, enabling research on disease risk in multiple populations. Further, this diversity supports broadly applicable results and can help uncover health disparities that occur due to disproportionate environmental exposures for certain populations.

#### Data provided to PEGS DREAM Challenge teams

PEGS DREAM Challenge teams were provided access to data from PEGS Data Freeze 3.1 for PEGS participants who completed the Health & Exposure Survey (N = 9,449) (see Table 1). The questionnaire-based data include information on demographics, health, environmental exposures, socioeconomic status, and lifestyle factors (Tables 1 and 2). Genomic data on SNVs and indel genotypes, structural variant calls, HLA genotypes, estimated aggregate telomeric content, and ancestry estimations were derived from WGS. Genome-wide methylation profiling data were obtained using the Infinium MethylationEPIC v1.0 BeadChip Kit (Figure 6). Geospatial data include exposure estimates and proximity to hazards calculated using geospatial linkages with various databases, linkages from the MERRA-2 project providing estimates of climate and environmental metrics from satellite observations, and SVI and EJI data (Table 3).

The dataset (N = 9,449) was filtered to remove outliers, related individuals determined from WGS data, and individuals with mismatched self-reported and WGS-inferred race (N = 9,184). The filtered data were split into individuals with and without WGS data. Each subset was then randomly split into thirds to create training (n = 3,062), validation (n = 3,062), and test (n = 3,060) subsets. Accordingly, the training, validation, and test subsets each consisted of approximately 1,500 individuals with and 1,500 individuals without sequencing data. The test subset was not shared with Challenge participants and was held out to score Final round submissions. Table 4 provides the demographics of participants included in the DREAM Challenge data.

In the test dataset, 983 (32.8%) PEGS participants self-reported a diagnosis of hypercholesterolemia in the Health & Exposure Survey. Table 4 shows the number of PEGS participants available for the specific PEGS data component. In the PEGS DREAM Challenge data, WGS data were available for 4,544 participants, and Internal and External Exposome Survey data were available for 2,722 and 3,173 participants, respectively.

#### Synthetic data

The synthpop v1.8 library in R was used to create synthetic data from the original tabular PEGS data. For each data file stratified into training/validation/testing sets, a synthetic version equal in size was created using a random forest model, sequentially for each feature. By generating synthetic data for each table separately, inter-table correlation was not preserved. However, the data were appropriate for developing methods that were validated during this Challenge phase. For large survey and genomic data tables, independent resampling and error injection were performed to match the data types in the original dataset but remove correlation between features.

The synthetic dataset contained training (_train.*) and validation (*_val.*) data that reflect the structure and sample sizes of the original PEGS dataset. In the synthetic dataset, “NA” was used for unmeasured missing phenotype variables to preserve the original data structure. The synthetic and original datasets contain the same number of individuals in each file.

Multiple measures were taken to protect the privacy of PEGS participants. First, all data were de-identified by removing all personally identifiable information (PII) and protected health information (PHI). Second, the data were anonymized by replacing participant IDs with generated IDs for each data component. Third, Challenge participants were not provided access to the original PEGS data. Only the synthetic data were available for download for model construction and code development. The submitted models were evaluated using the original PEGS data that were unavailable for download by Challenge participants.

### Challenge Details

The PEGS DREAM Challenge invited teams to develop models to identify the myriad factors affecting hypercholesterolemia and improve understanding of the etiology of this complex disease. Determining modifiable factors can help reduce the likelihood of developing hypercholesterolemia and its associated health complications. The Challenge was launched on May 1, 2024, and a Leaderboard round ran from May 10 to August 6, 2024. The Final round ran from August 6 to August 19, 2024. Winners were announced on August 31, 2024 and presented their models and findings at the Regulatory & Systems Genomics with DREAM Challenges Conference (RSGDREAM2024) in Madison, Wisconsin, USA (https://www.iscb.org/rsgdream2024/home).

Challenge teams could take part in one or both distinct tasks. The goal of Task 1was to classify individual hypercholesterolemia disease status in the held-out test dataset using combinations of the health, exposomic, geospatial, and genomic data available for the PEGS cohort. The goal of Task 2, an ideation challenge, was to develop novel hypotheses and models using the multi-dimensional PEGS data to improve understanding of the etiology of hypercholesterolemia beyond conventional clinical and genetic risk factors.

To ensure rigorous evaluation while safeguarding participant privacy, the Challenge was implemented using a model-to-data framework ^18^. In this paradigm, the PEGS cohort data were stored securely on the Challenge platform, and participants submitted containerized models that were executed against hidden validation datasets. This approach, successfully pioneered in previous DREAM Challenges, enabled participants to develop innovative methods without direct access to confidential health and genomic data while organizers ensured unbiased evaluation. This framework was used in previous Challenges that include the Digital Mammography DREAM Challenge (2017), Patient Mortality Prediction EHR DREAM Challenge (2019), COVID-19 EHR DREAM Challenge (2020), and CD2H NLP Sandbox (2021).

#### Challenge Task 1 – Disease Classification

Figure 4 shows the workflow for Task 1 of the PEGS DREAM Challenge. Participants were provided with synthetic data with the same format and structure as the original PEGS data. For details, see the synthetic data section. The training and validation subsets were available for model training and model optimization, respectively, and participants could combine or split the subsets in various proportions. The test dataset was not shared with challenge contributors and was used for scoring and evaluation.

Participants were asked to integrate the multi-dimensional components of PEGS, comprising health, exposure, genomic, and geospatial data, to create a model that surpassed the classification accuracy of an AUROC = 0.7358. The AUROC value was obtained from a model using a hypercholesterolemia PGS computed for PEGS participants using the weights and metadata from the hypercholesterolemia PGS by Weissbrod et al. ^6^ from the Polygenic Score Catalog ^7,8^ as the main predictor. This hypercholesterolemia PGS was chosen because it included the highest number of genomic variants in the score compared to other hypercholesterolemia scores in the catalog and included individuals from multiple ancestries in the evaluation set, mirroring the diversity of the PEGS cohort. A detailed description of PGS computation for PEGS participants is provided in Schaid et al. ^19^

Teams uploaded Docker containers and writeups in .docx or .pdf format to their Synapse Project workspace. The submitted Docker container produced a single output file classifying hypercholesterolemia status for the held-out test dataset in a two-column .csv file containing participant IDs and disease probability. The submitted Docker containers were run on the original PEGS data, with a limit of five model submissions per day in the Leaderboard round and one model submission per day in the Final round. Scoring was based on the AUROC of each submitted model in the validation subset for the Leaderboard round and in the test dataset for the Final round. AUPRC was employed as a tiebreaking metric.

#### Challenge Task 2 – Ideation Challenge

In Task 2, Challenge participants leveraged the comprehensive PEGS dataset to develop bold and novel hypotheses and models, integrating its health, exposure, geospatial, and genomic components to provide multi-dimensional insights. This challenge was aimed at harnessing collective creativity to generate a pipeline of fresh ideas to address complex challenges. Participants proposed novel research questions and methodologies, with the goals of fostering ideation related to emerging omics technologies and their applications, incentivizing outside-the-box thinking beyond established analytical approaches, and promoting collaboration and interdisciplinary approaches for holistic exploration.

Teams submitted a writeup in .docx or .pdf format documenting their hypotheses and models, which were scored by a panel of judges based on creativity, significance, interpretability, innovation, utility, feasibility, and potential translational impact. Submissions with working or detailed models or specific mathematical model descriptions were given higher scores. Participants were encouraged to submit models involving GxE interactions, genome-wide environment interaction studies (GWEIS), and similar methods. Innovation was emphasized to encourage participants to explore novel approaches.

#### Challenge Resources

The PEGS DREAM Challenge website (https://www.synapse.org/PEGS) describes in detail the PEGS cohort, data available to participants, and two Challenge tasks and provides submission guidelines and evaluation criteria. The website also outlines the Challenge timeline, participation criteria, and conditions for data use. Additionally, the AUROCs of the models submitted for Task 1 computed in the validation data were published live on the Challenge website during the Leaderboard round to provide participants with feedback and enable them to improve their models for the Final round.

In addition to the Challenge website, participants had access to several other PEGS resources for data exploration, hypothesis generation, and investigation of results of prior analyses in the PEGS data. The PEGS Explorer (https://pegsexplorer.niehs.nih.gov/) web application shares published results of ExWAS conducted in PEGS data and visualizations of the complex correlations among the exposures through correlation globes ^10,11^. Participants could explore and use these results for the Challenge. Participants could also request access to the i2b2 self-service web-based tool for data exploration, which enabled participants to explore de-identified and aggregated PEGS data by building queries. The results assisted with data exploration for model construction and hypothesis generation. Participants were also provided with access to code libraries for the PEGS data as a reference for development efforts. This included a library containing common utilitarian functions for ingesting and analyzing the PEGS data (https://github.com/fsakhtari/PEGS_common/blob/master/pegs_common_utils.R) and example scripts (https://github.com/nathanielmacnell/PEGStools) for working with the PEGS geospatial data.

All methods were carried out in accordance with relevant guidelines and regulations. The PEGS study protocol and/or the use of PEGS data for this study were approved by the NIEHS IRB, protocol 04-E-0053. Informed consent was obtained from all participants and/or their legal guardians.

## Supplementary Material

This is a list of supplementary files associated with this preprint. Click to download.

• PEGSDREAMChallengemanuscriptsupplement26092025.docx

## Figures and Tables

**Table 1.** PEGS data components. The various data components available in the PEGS cohort. Data reported from PEGS Data Freeze 3.1 created on 6/27/2023.

Category	Component	Description
Survey Data	Demographic and administrative data	Demographics, consent, addresses, and administrative data for all participants
	Health & Exposure Survey	Demographics, health, family history of disease, environmental exposures, socioeconomic status, and lifestyle factors
	External Exposome Survey (Exposome A)	Residential and occupational environmental exposures
	Internal Exposome Survey (Exposome B)	Medication use, physical activity, stress, sleep, diet, genetics, and reproductive history
Geospatial Data	Hazards data	Exposure estimates and proximity measures calculated using geospatial linkages from various databases
	MERRA-2 Data (Earthdata)	Geospatial data linkages from the MERRA-2 project containing consistent estimates of climate and environmental metrics from a range of satellite-based environmental observations
	Social Vulnerability Index (SVI) data	Geospatial data linkages for the CDC/ATSDR SVI containing summaries of social determinants of health at the census-tract level
	Environmental Justice Index (EJI) data	Geospatial data linkage for the CDC/ATSDR EJI containing summaries of environmental, social, and health factors at the census-tract level
Genomic data	Single nucleotide variants (SNVs)	SNV and small indel genotypes derived from the whole genome sequencing (WGS) data in PLINK’s .bed/.bim/.fam format
	Structural variants	Structural variant calls generated from the WGS data in .vcf format consisting of large deletions, duplications, and inversions
	Human leukocyte antigens (HLA) Genotypes	HLA genotypes identified from the WGS data for 20 HLA genes with up to six digits of specificity
	Telomeric content	Aggregate telomeric content estimated from WGS reads reported as telomeric reads per GC content-matched million reads
	Local and global ancestry estimations	Inferred local ancestry per chromosome after haplotype phasing and global estimates of percent ancestry for each participant
	Methylation data	Genome-wide methylation profiling data using the Infinium MethylationEPIC v1.0 BeadChip Kit targeting 866,297 CpG sites

**Table 2.** Categories of questions in the PEGS Health & Exposure Survey, External Exposome Survey, and Internal Exposome Survey. The table shows the high-level survey question categories of the surveys administered to PEGS participants. Data reported from PEGS Data Freeze 3.1 created on 6/27/2023.

Health & Exposure Survey		
About Your Family’s Health	Diabetes and Endocrine	Neurologic
About Your General Health	Digestive	Occupation
About Your Home Life	Exposures	Renal
About Your Mood	Fatigue	Reproductive (Females Only)
Bones, Joints, and Muscles	Hematological	Reproductive (Males Only)
Cancer	Immune	Respiratory
Cardiovascular	Lifestyle	Skin, Eyes, and Hair

External Exposome (Exposome A)		
Characteristics of Current and Past Residences: Agricultural Property Use Garage and Basement Heating and Cooling Pesticides and Insecticides Pets Surrounding Area Walls and Flooring Water and Dampness	Chemical and Metal Exposures at Work	
	Ultraviolet Light Exposure	
	Workplace Characteristics	
Hobby Exposures		

Internal Exposome (Exposome B)		
Chemotherapy/Radiation Therapy	Physical Activity	
Dietary Behavior	Reproductive History (Females Only)	
Dietary Intake	Sleep	
Genetic History	Stress	
Infectious Disease	Vitamins, Minerals, and Other Supplement Use	
Medications	Twin/Triplet Siblings and Birth Order	
Other		

**Table 3.** Summary of PEGS geospatial data. An overview of the geocoding and GIS data linkages available in the PEGS cohort. Data reported from PEGS Data Freeze 3.1 created on 6/27/2023.

Source	Description	Examples
Geocodes (GIS)	Geocoded data from multiple participant-provided addresses at the time of initial enrollment, completion of the Health & Exposure Survey, completion of the External Exposome Survey, the longest-lived childhood address, and the longest-lived adulthood address from the External Exposome Survey	Geographic coordinates (latitude and longitude) from multiple participant-provided addresses
Hazards	Exposure estimates computed from Department of Transportation (DOT) data	Information on train tracks, rail depots, and roadways, such as total major roadway length and distance to the nearest rail depot
Hazards	Exposure estimates computed from Federal Aviation Administration (FAA) data	Information on aircraft departure and arrival sites (e.g., distance to the nearest airport)
Hazards	Exposure estimates computed from Federal Communications Commission (FCC) data	Information on cellular network towers (e.g., nearest cell tower)
Hazards	Exposure estimates computed from the North Carolina Department of Environmental Quality (NCDEQ)	Distance to multi-pollutant point sources such as swine caged feeding operations (CAFOs), hazardous waste sites, hazardous spill sites, EPA superfund sites, and wastewater treatment plant release sites
Hazards	Exposure estimates computed from Nuclear Regulatory Commission (NRC) data	Distance to nuclear power stations
Hazards	Exposure estimates computed from Atmospheric Composition and Analysis Group (ACAG) data	Particulate matter concentrations such as PM2.5 total, PM2.5 sulfate, and PM2.5 black carbon and other
Hazards	Exposure estimates computed from Center for Air, Climate, and Energy Solutions (CACES) data	Concentrations for multiple pollutants such as carbon monoxide, nitrogen dioxide, and ozone concentration
Hazards	Exposure estimates computed from Toxics Release Inventory (TRI) data	Emissions for chemicals of interest such as benzene, ethylbenzene, xylene, and toluene
MERRA-2 data (Earthdata)	Geospatial data linkages from the Modern Era Retrospective Analysis for Research and Applications (MERRA-2) project to assimilate a range of satellite-based environmental observations into a consistent estimate of climate and environmental metrics	Particulate, gas, meteorological, and health-relevant exposure indicators such as dust sedimentation, organic carbon emission bin, SO_2_ biomass burning emissions, and sea-level pressure
Social Vulnerability Index (SVI)	Geospatial data linkages for CDC/ATSDR SVI designed to consistently quantify multiple social determinants of health across the United States over time	Summaries of social determinants of health at the census-tract level, including an overall index, four component indices (socioeconomic status, household characteristics, racial and ethnic minority status, and housing type/transportation), and source variables used to compute each index component (e.g., poverty, education, overcrowding, access to a vehicle)
Environmental Justice Index (EJI)	Geospatial data linkages for CDC/ATSDR EJI containing summaries and ranks of the cumulative impacts of environmental injustice on health at the census-tract level	Ranks for each census tract based on 36 environmental, social, and health factors grouped into 10 domains and three overarching modules: environmental burden, social vulnerability, and health vulnerability

**Table 4.** PEGS DREAM Challenge participant demographics and data availability. Demographics of participants and survey and WGS data availability in the PEGS DREAM Challenge data in the training, validation, and test subsets. Age was computed at the time of completion of the Health & Exposure Survey. The PEGS DREAM Challenge data was created from PEGS Data Freeze 3.1 created on 6/27/2023.

Demographic variable	Training data n (%)	Validation data n (%)	Test data n (%)
**Total N**	3062 (33.3)	3062 (33.3)	3060 (33.3)

**Sex**			
Male	994 (32.5)	993 (32.4)	1039 (34)
Female	2068 (67.5)	2069 (67.6)	2021 (66)

**Race**			
Other	133 (4.3)	131 (4.3)	140 (4.6)
Black or African American	696 (22.7)	678 (22.1)	620 (20.3)
White	2157 (70.4)	2179 (71.2)	2214 (72.4)
NA	76 (2.5)	74 (2.4)	86 (2.8)

**Ethnicity**			
Non-Hispanic/Non-Latino	2886 (94.3)	2887 (94.3)	2905 (94.9)
Hispanic/Latino	119 (3.9)	130 (4.2)	112 (3.7)
NA	57 (1.9)	45 (1.5)	43 (1.4)

**Education**			
12th grade or less	528 (17.2)	493 (16.1)	501 (16.4)
College, technical or vocational	887 (29)	915 (29.9)	912 (29.8)
Bachelor's degree	828 (27)	811 (26.5)	838 (27.4)
Graduate or professional degree	800 (26.1)	819 (26.7)	783 (25.6)
NA	19 (0.6)	24 (0.8)	26 (0.8)

**Income**			
Less than $20,000	432 (14.1)	400 (13.1)	417 (13.6)
$20,000 to 49,999	896 (29.3)	937 (30.6)	907 (29.6)
$50,000 to 79,999	740 (24.2)	728 (23.8)	718 (23.5)
$80,000 or more	892 (29.1)	889 (29)	906 (29.6)
NA	102 (3.3)	108 (3.5)	112 (3.7)

**Age in years (SD)**	50 (16)	50.4 (15.9)	50.2 (16)

**Health & Exposure Survey completed**			
N=	3062 (100)	3062 (100)	3060 (100)

**External Exposome completed**			
No	2001 (65.3)	1992 (65.1)	2018 (65.9)
Yes	1061 (34.7)	1070 (34.9)	1042 (34.1)

**Internal Exposome completed**			
No	2156 (70.4)	2151 (70.2)	2155 (70.4)
Yes	906 (29.6)	911 (29.8)	905 (29.6)

**WGS available**			
No	1547 (50.5)	1547 (50.5)	1546 (50.5)
Yes	1515 (49.5)	1515 (49.5)	1514 (49.5)

**Hypercholesterolemia status**			
Yes	1018 (33.2)	1011 (33.0)	983 (32.1)
No	2012 (65.7)	2001 (65.3)	2029 (66.3)

## Data Availability

The PEGS dataset analyzed in this study is not publicly available because it contains sensitive human participant data and is subject to ethical and privacy restrictions. De-identified data supporting the findings of this study are available from the corresponding author upon reasonable request, subject to review and approval by the NIEHS IRB, compliance with applicable ethical and legal requirements, and execution of any required data use agreement. Requests for access should be directed to alison.motsinger-reif@nih.gov or through a web form inquiry: https://www.niehs.nih.gov/research/atniehs/labs/crb/studies/pegs/collaboration/proposal. PEGS DREAM Challenge participants were required to provide code as Docker containers to run their models for evaluation. Code libraries for working with PEGS data were provided as resources (e.g., https://github.com/fsakhtari/PEGS_common/blob/master/pegs_common_utils.R, https://github.com/nathanielmacnell/PEGStools).
